# Trends in Tobacco Smoke Exposure and Blood Lead Levels Among Youths and Adults in the United States: The National Health and Nutrition Examination Survey, 1999–2008

**DOI:** 10.5888/pcd10.130056

**Published:** 2013-12-19

**Authors:** Patricia A. Richter, Ellen E. Bishop, Jiantong Wang, Rachel Kaufmann

**Affiliations:** Author Affiliations: Ellen E. Bishop, Jiantong Wang, RTI International, Atlanta, Georgia; Rachel Kaufmann, Centers for Disease Control and Prevention, Atlanta, Georgia.

## Abstract

**Introduction:**

Tobacco smoke is a source of exposure to thousands of toxic chemicals including lead, a chemical of longstanding public health concern. We assessed trends in blood lead levels in youths and adults with cotinine-verified tobacco smoke exposure by using 10 years of data from the National Health and Nutrition Examination Survey.

**Methods:**

Geometric mean levels of blood lead are presented for increasing levels of tobacco smoke exposure. Regression models for lead included age, race/ethnicity, poverty, survey year, sex, age of home, birth country, and, for adults, alcohol consumption. Lead levels were evaluated for smokers and nonsmokers on the basis of age of residence and occupation.

**Results:**

Positive trend tests indicate that a linear relationship exists between smoke exposure and blood lead levels in youths and adults and that secondhand smoke exposure contributes to blood lead levels above the level caused by smoking.

**Conclusion:**

Youths with secondhand smoke exposure had blood lead levels suggestive of the potential for adverse cognitive outcomes. Despite remediation efforts in housing and the environment and declining smoking rates and secondhand smoke exposure in the United States, tobacco smoke continues to be a substantial source of exposure to lead in vulnerable populations and the population in general.

## MEDSCAPE CME

Medscape, LLC is pleased to provide online continuing medical education (CME) for this journal article, allowing clinicians the opportunity to earn CME credit.

This activity has been planned and implemented in accordance with the Essential Areas and policies of the Accreditation Council for Continuing Medical Education through the joint sponsorship of Medscape, LLC and Preventing Chronic Disease. Medscape, LLC is accredited by the ACCME to provide continuing medical education for physicians.

Medscape, LLC designates this Journal-based CME activity for a maximum of 1 **AMA PRA Category 1 Credit(s)™**. Physicians should claim only the credit commensurate with the extent of their participation in the activity.

All other clinicians completing this activity will be issued a certificate of participation. To participate in this journal CME activity: (1) review the learning objectives and author disclosures; (2) study the education content; (3) take the post-test with a 70% minimum passing score and complete the evaluation at www.medscape.org/journal/pcd (4) view/print certificate.

**Release date: December 18, 2013; Expiration date: December 18, 2014**

### Learning Objectives

Upon completion of this activity, participants will be able to:

- Evaluate the effects of secondhand smoke (SHS) exposure on blood lead levels (BLLs) among children and adolescents
- Analyze the potential for additive effects of smoking and SHS exposure on BLLs
- Distinguish the group with the highest average BLLs in the current study
- Assess temporal trends in BLLs among children and adults

**EDITORS**

Caran Wilbanks, Editor, *Preventing Chronic Disease*. Disclosure: Caran Wilbanks has disclosed no relevant financial relationships.

**CME AUTHOR**

Charles P. Vega, MD, Associate Professor and Residency Director, Department of Family Medicine, University of California, Irvine. Disclosure: Charles P. Vega, MD, has disclosed no relevant financial relationships.

**AUTHORS AND CREDENTIALS**

Disclosures: Patricia A. Richter, Ellen E. Bishop, Jiantong Wang, and Rachel Kaufmann have disclosed no relevant financial relationships.

Affiliations: Patricia A. Richter, Office of Smoking and Health, Centers for Disease Control and Prevention, Atlanta, Georgia; Ellen E. Bishop and Jiantong Wang, Chronic & Infectious Disease Research Program, RTI International, Atlanta, Georgia; and Rachel Kaufmann, Office of Surveillance, Epidemiology, and Laboratory Services, Centers for Disease Control and Prevention, Atlanta, Georgia.

## Introduction

Tobacco use is the largest preventable cause of disease, disability, and death in the United States. Each year, an estimated 443,000 people die prematurely from smoking or exposure to secondhand smoke (SHS) and another 8.6 million live with a serious illness caused by smoking (1).

Smoking harms nearly every organ in the body, causing many diseases and reducing the health of smokers in general (2). There is no safe level of exposure to SHS. In adults, SHS exposure causes lung cancer and cardiovascular disease. Infants and young children with SHS exposure have increased risk for sudden infant death syndrome, lower respiratory tract infections, decreased lung function, and other adverse health outcomes (3).

Cigarette smoke contains more than 7,000 chemicals. Many are carcinogens and toxicants implicated in major diseases (4–7). Active and passive smoking are sources of exposure to metals (8–11). Lead is of particular public health concern because it accumulates in the body. The Centers for Disease Control and Prevention (CDC) has concluded that no level of lead in a child’s blood can be specified as safe (12–14).

Smoking rates and SHS exposure have been declining in the United States (15,16). Consequently, we examined trends in blood lead levels (BLLs) in youths and adults with cotinine-verified tobacco smoke exposure from 1999 to 2008. Specific questions addressed in this analysis are 1) What are current BLLs in US children and adults with exposure to tobacco smoke? and 2) Are changes over time in BLLs among youths and adults exposed to tobacco smoke distinguishable from BLLs among smoke-free individuals with consideration of other environmental influences such as lead exposure in the home and occupational exposure to metals?

## Methods

All data were obtained from the CDC’s National Health and Nutrition Examination Survey (NHANES) (www.cdc.gov/nchs/nhanes.htm). NHANES is representative of civilian, noninstitutionalized residents of the United States aged 2 months or older. Five cycles of data were included: 9,965 participants in NHANES 1999–2000, 11,039 in NHANES 2001–2002, 10,122 in NHANES 2003–2004, 10,348 in NHANES 2005–2006, and 10,149 in NHANES 2007–2008 (total = 51,623). In the 5 NHANES cycles in this analysis, there were 43,627 participants who were at least 3 years of age — the minimum age for measuring cotinine — and who completed the health examination at a NHANES mobile examination center (MEC). Only data from respondents with cotinine and lead measurements were included in the analysis. For participants aged 12 or older, the analysis was further limited to those who responded to smoking questions. Smoking questions are asked only of respondents aged 12 years or older. The final analysis file contained data on the 34,154 respondents with the required laboratory measurements and a validated smoking status.

Serum cotinine was measured by a high-performance liquid chromatography/atmospheric pressure ionization tandem mass spectrometry method as described previously (17). Whole blood lead was determined by using inductively coupled plasma mass spectrometry (18).

*Nonsmokers* were initially identified as respondents who answered “no” when asked about use in the past 5 days of any product containing nicotine (cigarettes, pipes, cigars, chewing tobacco, snuff, nicotine patches, nicotine gum, or any other product containing nicotine). Any nonsmoker who answered “yes” to the question “Have you smoked at least 100 cigarettes in your life?” and answered “Every day” and “Some days” to “Do you now smoke cigarettes?” was removed from the analysis. Any nonsmoker with a cotinine measurement of 10 nanograms per milliliter (ng/mL) or greater, a level consistent with active smoking, was excluded as described previously (10). Any participant younger than 12 years was considered a nontobacco user, but was excluded if their cotinine was 10 ng/mL or greater. Therefore, a *validated nonsmoker* was a self-reported nonsmoker (or a participant younger than 12) with a cotinine measurement of less than 10 ng/mL. There were 652 self-reported nonsmokers excluded from the final analytic sample because they had cotinine levels 10 ng/mL or greater.

*Smokers* were respondents 12 years of age or older who answered “yes” when asked about use in the past 5 days of any product containing nicotine (cigarettes, pipes, cigars, chewing tobacco, snuff, nicotine patches, nicotine gum, or any other product containing nicotine) but who had not used noncigarette sources of nicotine (pipes, cigars, chewing tobacco, snuff, or other nicotine products) in the past 5 days. Smokers who used noncigarette sources of nicotine were excluded. We excluded 825 self-reported smokers from the final analytic sample because they had cotinine levels less than 10 ng/mL.

As previously reported (10), we used an estimated value of 0.035 ng/mL for cotinine levels below the level of detection. The geometric mean (GM) of cotinine in this population, 0.256 ng/mL, was chosen as the cutoff point to define “lower” (ie, below the mean) and “higher” (ie, above the mean) SHS exposure (10). Tobacco smoke exposure was categorized into 4 levels: self-identified, unexposed nonsmokers (nonsmokers with cotinine ≤0.035 ng/mL); nonsmokers with lower SHS exposure (nonsmokers with cotinine >0.035 ng/mL and ≤0.256 ng/mL); nonsmokers with higher SHS exposure (nonsmokers with cotinine >0.256 ng/mL and ≤10 ng/mL); and smokers (self-identified cigarette smokers with cotinine >10 ng/mL).

Findings for participants who described themselves as non-Hispanic white, non-Hispanic black, or Mexican American are presented. Other races/ethnicities were included in total population estimates but their data are not presented separately because of the small number of participants in this category. Age was categorized into 7 groups (3–5 y, 6–11 y, 12–18 y, 19–34 y, 35–49 y, 50–64 y, and ≥65 y).

The poverty-to-income ratio (PIR) is the ratio between family income and the government-defined poverty threshold, which is based on income thresholds that vary by family size and composition and that are updated annually for inflation with the Consumer Price Index (19). The PIR was used to create a dichotomous variable to determine household income greater than or equal to (≥1.00) or below (<1.00) the poverty threshold. The poverty threshold was used as an indicator of socioeconomic status.

Age of residence was determined by the question “When was this {mobile home/house/building} originally built?” with categorical responses coded as 1949 or earlier, 1950 through 1977, or 1978 or later. Birth country was determined by the question “In what country {were you/was [sample person]} born?” Responses were categorized as United States or outside the United States. Answers to questions about frequency of drinking alcohol within the past 12 months were used to calculate days of drinking per year.

GM BLLs and occupation of participants 19 years of age or older were used to create 3 categories: “higher” lead exposure jobs, “lower” lead exposure jobs, and “currently unemployed.” Participant NHANES occupation codes were matched to industry subsectors identified in the National Institute for Occupational Safety and Health’s Adult Blood Lead Epidemiology and Surveillance (ABLES) program. NHANES job categories that matched ABLES industry subsectors in which adults are found to have BLLs of 25 micrograms per deciliter (µg/dL) or higher were categorized as “higher” lead jobs. Other NHANES job categories were categorized as “lower” lead jobs. Participants reporting being unemployed at the time of the survey were assigned to the “currently unemployed” category.

The unweighted sample by demographic characteristic is presented in Table 1.

**Table 1:** Sample Size by Demographic Characteristic for Lead and Cotinine Exposure, National Health and Nutrition Examination Surveys, 1999–2008

Demographic Characteristics	No.^a^
**Total**	34,154
**Age, y**	
3–5	1,930
6–11	4,538
12–18	7,059
19–34	5,829
35–49	4,955
50–64	4,552
≥65	5,291
**Race/ethnicity**	
Non-Hispanic white	13,881
Non-Hispanic black	8,020
Mexican American	8,978
Other	3,275
**Sex**	
Male	16,318
Female	17,836
**Poverty-to-income ratio (PIR)^b^**	
Below the PIR	7,763
Greater than or equal to the PIR	23,984

a Numbers may not equal total because of missing data.

b The poverty-to-income ratio (PIR) is the ratio between family income and the government-defined poverty threshold, which is based on income thresholds that vary by family size and composition and that are updated annually for inflation with the Consumer Price Index (19). The PIR was used to create a dichotomous variable to determine household income greater than or equal to (≥1.00) or below the poverty threshold (<1.00).

### Statistical methods

Sampling weights provide unbiased national population estimates that adjust for unequal probabilities of selection, adjust to independent population controls, and account for nonresponses. The weights were poststratified to the US population as estimated by the Census Bureau. Because we used multiple data cycles, we calculated analytic survey weight following the NHANES documentation provided on its website. When combining 2 or more cycles of NHANES data, a new variable is created by assigning a fraction of the year weight based on how many years a person was sampled out of the total number of sample years. For this study, this method involved taking two-fifths of the special 4-year MEC weight for 1999 through 2002 and one-fifth of the 2-year MEC weight for 2003–2004, 2005–2006, and 2007–2008. For analyses, we used SAS (SAS Institute, Inc, Cary, North Carolina) and SUDAAN (Research Triangle Institute, Research Triangle Park, North Carolina), a program that adjusts for complex sample design when variance estimates are calculated.

Initially, estimates for GM with 95% confidence intervals were calculated for lead by demographic characteristic and for each exposure level. Then linear regression was used to determine if BLLs, by age, race, or sex, were significantly different by smoke exposure, with log transformations of BLL as the dependent variable and SHS or smoking (smoke exposure) as the independent variable. If an overall difference was significant by smoke exposure, then *t* tests were performed to identify the specific difference(s). The specific differences tested were “no exposure versus lower exposure,” “lower exposure versus higher exposure,” “higher exposure versus smoker,” and “no exposure versus smoker.” For children aged 3 to 11 years, the comparisons were between “no exposure versus lower exposure,” “lower exposure versus higher exposure,” and “no exposure versus higher exposure.” To limit type I errors on multiple comparisons, Bonferroni correction was used.

### Trend test

Both linear and quadratic trend tests were used to examine dose–response relationships in increasing smoke exposures and the lead GM. To examine trends among nonsmokers, smokers were removed from the analysis.

### Regression models

Regression models were used to further investigate several factors related to BLLs. The base model included survey year and smoke exposure level. Other predictors were added individually to the base model. The youth models added age group, race/ethnicity, sex, poverty, age of home, and birth country. Occupation was not included in the adult model because occupation data were available only for 1999 through 2004.

After assessing the variables individually, full models were created with all variables. An interaction term between survey year and smoke exposure level was added to both full models; this interaction term was not significant at the .05 level in either model.

The full youth model is

Log of BLL = survey year + smoke exposure level + age group + race/ethnicity + sex + poverty + age of home + birth country + (survey year × smoke exposure level)

The full adult model is

Log of BLL = survey year + smoke exposure level + age group + race/ethnicity + sex + poverty + age of home + birth country + days/year drinking + (survey year × smoke exposure level)

Before fitting the model, correlations among the youth predictors or adult predictors for each model were examined. None of their correlation coefficients was above 0.05. Also, variance inflation factors (VIF) were calculated to determine how much of the increase in the variance of the estimated regression coefficients was due to collinearity. All youth and adult VIF values were below the common cutoff of 5, so all independent variables were retained.

## Results

The results for the full regression models are in Table 2. All predictor variables in the youth model were significant at the .05 level. In the full adult model, the poverty variable was significant (versus nonsignificant in the individual model) (Table 2). The race/ethnicity variable became highly significant in the full model rather than marginally significant in the individual model. After the age variable was removed from the adult model, the poverty variable became nonsignificant. After removing age, birth country, and days of drinking per year variables, both poverty and race/ethnicity variables became nonsignificant.

**Table 2:** Regression Models on the Measure of Lead Level for Youths (Aged 3–11 Years) and Adults (Aged 19 Years or Older), National Health and Nutrition Examination Surveys, 1999–2008

Variables	Youth β, SE β	Youth *P* Value	Adult β, SE β	Adult *P* Value
**Age, y**		<.001		<.001
3–5	0.27, 0.02	<.001	NA	NA
6–11	0.00, 0.00	Reference	NA	NA
19–34	NA	NA	−0.81, 0.02	<.001
35–49	NA	NA	−0.50, 0.02	<.001
50–64	NA	NA	−0.18, 0.02	<.001
≥65	NA	NA	0.00, 0.00	Reference
**Race/ethnicity**		<.001		<.001
Non-Hispanic white	0.00, 0.00	Reference	0.00, 0.00	Reference
Non-Hispanic black	0.32, 0.03	<.001	0.13, 0.02	<.001
Mexican American	0.10, 0.04	<.006	0.09, 0.03	.004
Other	0.03, 0.03	.45	0.01, 0.03	.86
**Sex**		<.001		<.001
Male	0.07, 0.02	<.001	0.32, 0.01	<.001
Female	0.00, 0.00	Reference	0.00, 0.00	Reference
**Poverty to income ratio (PIR)^a^**		<.001		.008
Below PIR	0.19, 0.03	<.001	−0.06, 0.02	.008
Greater than or equal to the PIR	0.00, 0.00	Reference	0.00, 0.00	Reference
**Age of home**		<.001		<.001
1949 or earlier	0.31, 0.04	<.001	0.15, 0.02	<.001
1950–1977	0.08, 0.03	<.004	0.02, 0.01	<.173
1978 or later	0.00, 0.00	Reference	0.00, 0.00	Reference
**Birth country**		.001		<.001
United States	0.00, 0.00	Reference	0.00, 0.00	Reference
Outside United States	0.23, 0.07	.001	0.19, 0.02	<.001
**Days per year drinking alcohol**	NA	NA	0.00, 0.00	<.001

Abbreviations: NA, not applicable; SE, standard error.

a The poverty-to-income ratio (PIR) is the ratio between family income and the government-defined poverty threshold, which is based on income thresholds that vary by family size and composition and that are updated annually for inflation with the Consumer Price Index (19). The PIR was used to create a dichotomous variable to determine household income greater than or equal to (≥1.00) or below the poverty threshold (<1.00).

For combined nonsmoker and smoker participants 12 and older, significant linear and quadratic trend tests indicate that BLLs increase with increasing smoke exposure in a linear quadratic manner (concave upward dose–response relationship) in that the unit response increases with increasing exposure. Significant linear and nonsignificant quadratic trends among only nonsmokers (≥3 y) indicate that SHS exposure is positively related to BLLs in a supra-linear manner (Table 3).

**Table 3:** Geometric Mean Lead Levels^a^ Overall and by Level of Smoke Exposure by Demographic Characteristic, National Health and Nutrition Examination Surveys, 1999–2008^b^

Demographic Characteristic	Overall Geometric Mean Level, µg/dL	Nonsmokers			Smokers	Nonsmokers and Smokers, *P* Values			Nonsmokers Only, *P* Values		
		Geometric Mean Level Without SHS Exposure, µg/dL	Geometric Mean Level With Lower SHS Exposure, µg/dL	Geometric Mean Level With Higher SHS Exposure, µg/dL	Geometric Mean Level, µg/dL	Overall	Quadratic Trend	Linear Trend	Overall	Quadratic Trend	Linear Trend
**Age, y**											
3–5	1.57 (1.48–1.66)	1.24 (1.15–1.34)^c^	1.55 (1.43–1.67)^c^	2.06 (1.88–2.23)^c^	NA	NA			<.001	.5195	<.001
6–11	1.18 (1.13–1.23)	0.98 (0.94–1.02)^c^	1.20 (1.14–1.26)^c^	1.53 (1.43–1.63)^c^	NA	NA			<.001	.4652	<.001
12–18	0.88 (0.85–0.91)	0.78 (0.75–0.81)^c^	0.92 (0.88–0.97)^d^	1.02 (0.97–1.07)	0.93 (0.87–0.99)^c^	<.001	<.001	<.001	<.001	.1465	<.001
19–34	1.07 (1.04–1.10)	0.87 (0.84–0.90)^c^	1.00 (0.94–1.06)	1.04 (0.99–1.09)^c^	1.42 (1.36–1.48)^c^	<.001	.001	<.001	<.001	.1303	<.001
35–49	1.42 (1.37–1.46)	1.15 (1.11–1.19)^c^	1.40 (1.31–1.48)	1.45 (1.33–1.57)^c^	1.97 (1.88–2.06)^c^	<.001	.036	<.001	<.001	.0146	<.001
50–64	1.84 (1.79–1.89)	1.60 (1.54–1.66)^c^	1.77 (1.70–1.85)	1.92 (1.75–2.09)^c^	2.50 (2.40–2.59)^c^	<.001	.002	<.001	<.001	.6853	<.001
≥65	2.08 (2.03–2.13)	1.97 (1.91–2.04)	2.08 (1.97–2.18)^e^	2.38 (2.21–2.54)	2.62 (2.45–2.78)^c^	<.001	.336	<.001	<.001	.2349	<.001
**Race/ethnicity**											
Non-Hispanic white	1.36 (1.33–1.39)	1.22 (1.18–1.26)^c^	1.33 (1.28–1.38)	1.35 (1.28–1.41)^c^	1.75 (1.70–1.80)^c^	<.001	<.001	<.001	<.001	.0532	<.001
Non-Hispanic black	1.56 (1.49–1.63)	1.25 (1.18–1.31)^c^	1.39 (1.31–1.48)^c^	1.63 (1.54–1.72)^c^	2.27 (2.17–2.36)^c^	<.001	<.001	<.001	<.001	.3031	<.001
Mexican American	1.42 (1.37–1.47)	1.26 (1.22–1.31)^c^	1.52 (1.44–1.60)	1.50 (1.38–1.62)^c^	2.05 (1.90–2.20)^c^	<.001	.009	<.001	<.001	.0001	<.001
**Sex**											
Male	1.66 (1.62–1.70)	1.43 (1.38–1.47)^c^	1.63 (1.57–1.68)	1.68 (1.61–1.74)^c^	2.18 (2.11–2.25)^c^	<.001	<.001	<.001	<.001	.0068	<.001
Female	1.18 (1.16–1.21)	1.10 (1.07–1.13)^e^	1.15 (1.11–1.19)	1.20 (1.14–1.25)^c^	1.48 (1.43–1.54)^c^	<.001	<.001	<.001	.001	.9617	<.001
**Poverty-to-income ratio (PIR)^f^**											
Below the PIR	1.51 (1.45–1.57)	1.30 (1.22–1.37)	1.35 (1.26–1.44)^c^	1.57 (1.47–1.66)^c^	1.82 (1.73–1.91)^c^	<.001	.016	<.001	<.001	.0931	<.001
Greater than or equal to the PIR	1.35 (1.32–1.38)	1.21 (1.17–1.24)^c^	1.34 (1.30–1.39)	1.35 (1.30–1.41)^c^	1.80 (1.74–1.85)^c^	<.001	<.001	<.001	<.001	.0025	<.001

Abbreviations: NA, not applicable.

a We used an estimated value of 0.035 ng/mL for cotinine levels below the level of detection. The geometric mean of cotinine in this population, 0.256 ng/mL, was chosen as the cutoff point to define “lower” (ie, below the mean) and “higher” (ie, above the mean) SHS exposures (10). Tobacco smoke exposures were categorized into 4 levels: self-identified, unexposed nonsmokers (nonsmokers with cotinine ≤0.035 ng/mL); nonsmokers with lower SHS exposure (nonsmokers with cotinine >0.035 ng/mL and ≤0.256 ng/mL); nonsmokers with higher SHS exposure (nonsmokers with cotinine >0.256 ng/mL and ≤10 ng/mL); and smokers (self-identified cigarette smokers with cotinine >10 ng/mL).

b For statistical analysis of differences in mean blood lead levels the following *t* test comparisons were performed: nonsmokers without SHS exposure and nonsmokers with lower SHS exposure; nonsmokers with lower SHS exposure and nonsmokers with higher SHS exposure; nonsmokers with higher SHS exposure and smokers; smokers and nonsmokers without SHS exposure. For children aged 3 to 11, comparisons were nonsmokers without SHS exposure and nonsmokers with lower SHS exposure, nonsmokers with lower SHS exposure and nonsmokers with higher SHS exposure, and nonsmokers without SHS exposure and nonsmokers with higher SHS exposure.

c *P* < .001.

d *P* < .01.

e *P* < .05.

f The poverty-to-income ratio (PIR) is the ratio between family income and the government-defined poverty threshold, which is based on income thresholds that vary by family size and composition and that are updated annually for inflation with the Consumer Price Index (19). The PIR was used to create a dichotomous variable to determine household income greater than or equal to (≥1.00) or below the poverty threshold (<1.00).

Trend test results indicate that for youths aged 3 to 11 BLLs increase with increasing smoke exposure but with some fluctuation (Table 3). Lead GM in the youngest participants (aged 3–5 y) was 1.25-fold higher for those with lower SHS exposure and 1.7-fold higher for those with higher SHS exposure compared with the youngest participants without SHS exposure.

Among all participants 12 years or older, the highest BLLs were seen in older smokers (Table 3). Non-Hispanic black and Mexican American participants had higher mean BLLs than did whites in both the youth and adult models. Men overall, and in all exposure groups, had higher BLLs than did women. Nonsmoking participants below the poverty threshold without SHS exposure and with higher SHS exposure had higher mean BLLs than did the corresponding groups at or above the poverty threshold. However, there was no difference in BLLs of smokers above and smokers below the poverty threshold.

**Figure 1:**
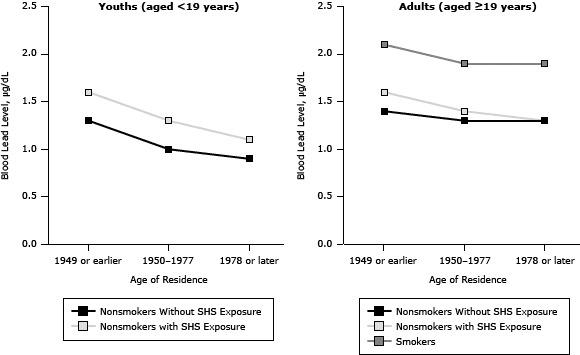
Blood lead levels in youths with and without exposure to secondhand smoke (SHS) and in adult smokers and nonsmokers with and without SHS exposure, by categories of age of residence, National Health and Nutrition Examination Surveys, 1999–2008. **Table:** 

People living below the poverty threshold, people living in homes built in 1949 or earlier (Figure 1), or people born outside the United States had higher BLLs (Table 2). For youths, there was a significant decreasing trend for BLLs by survey year. Nonsmoking youths with SHS exposure had higher BLLs than did the group without SHS exposure in all survey years. For adults, there was again a significant decreasing trend for BLLs by survey year. The BLLs in adult smokers (19 years of age or older) were higher than the BLLs for adult nonsmokers with or without SHS exposure (Table 3).

Age of Residence	Blood Lead Level, μg/dL				
	Youths (aged <19 years)		Adults (aged ≥19 years)		
	Nonsmokers Without SHS Exposure	Nonsmokers With SHS Exposure	Nonsmokers Without SHS Exposure	Nonsmokers With SHS Exposure	Smokers
1949 or earlier	1.3	1.6	1.4	1.6	2.1
1950–1977	1.0	1.3	1.3	1.4	1.9
1978 or later	0.9	1.1	1.3	1.3	1.9

**Figure 2:**
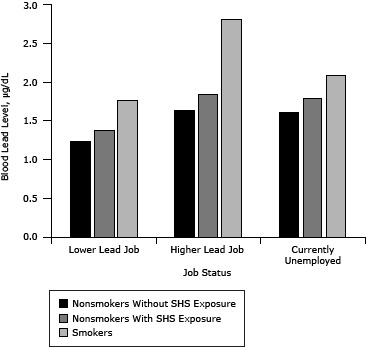
Blood lead levels among adults aged 19 or older, National Health and Nutrition Examination Surveys (NHANES), 1999–2008. Participant NHANES occupation codes were matched to industry subsectors identified in the National Institute for Occupational Safety and Health’s Adult Blood Lead Epidemiology and Surveillance (ABLES) survey. NHANES job categories that matched ABLES industry subsectors in which adults are found to have BLLs ≥25 micrograms per deciliter (µg/dL) were categorized as “higher” lead jobs. Other NHANES job categories were categorized as “lower” lead jobs. Participants reporting being unemployed at the time of the survey were assigned to the “currently unemployed” category. **Table:** 

After controlling for exposure to heavy metals in some occupations, higher BLLs are apparent for both smokers and nonsmokers with SHS exposure than for those not exposed (Figure 2).

Job Status	Blood Lead Level, μg/dL		
	Nonsmokers Without SHS Exposure	Nonsmokers With SHS Exposure	Smokers
Lower lead job	1.24	1.38	1.78
Higher lead job	1.64	1.85	2.83
Currently unemployed	1.61	1.80	2.09

Lead levels decreased among youths residing in newer homes (Table 2, Figure 1). Youths aged 3 to 5, males, those below the poverty threshold, or those born outside the United States had higher BLLs than did older children, females, those above the poverty threshold, or those born in the United States.

## Discussion

In 2010, nearly 1 in 5 US adults (45.3 million) were current smokers (16). In addition, an estimated 88 million nonsmokers, including 54% of children aged 3 to 11 years, were exposed to SHS (1). There is broad scientific agreement about which chemicals in conventional tobacco-burning cigarettes could be harmful to health (5). Relationships between smoking and body burdens of lead were noted in the public health literature decades ago (20). Our results confirm and extend prior findings to more recent years.

In this study, children aged 3 to 5 years had BLLs markedly higher than did older children (aged 12–18 y) and young adults (aged 19 to 34 y) despite the fact that BLLs reflect not only current exposures but also lead stored in the body (21). BLLs in SHS-exposed youths are higher than levels in unexposed youths, even when accounting for age of residence and other potential confounders.

The effect of low-level lead exposure on neurodevelopment has recently been reviewed (22). Decreased IQ and cognition have been observed in children 1 to 5 years old with BLLs of about 2 µg/dL (23). A study of SHS-exposed school-aged children reported poorer neurocognitive performance and hyperactivity symptoms at a mean BLL of 1.9 µg/dL (24). The lead GM of the youngest participants with SHS exposure in our study was 2 µg/dL, a level suggestive of the potential for adverse cognitive outcomes.

We took several measures to calculate unbiased estimates and to avoid overstating significance levels. Sampling weights were used to minimize any biases because of nonparticipation (18). Because BLLs are highly skewed, GMs were used in place of arithmetic means to minimize the effect of higher values, and significance tests were performed on the log-transformed data. The potential for misclassification based on incorrect reporting of smoking status or the half-life of cotinine was minimized by using both measures to define status instead of just one.

Our study is subject to several possible limitations. First, because our nonsmoker definition consisted of those that reported not smoking in the last 5 days, we may have included recent quitters and occasional smokers (25). Dual characterization of nonsmoker status by self-report and serum cotinine eliminated those with cotinine greater than 10 ng/mL. If the cotinine measurement condition were omitted from our nonsmoker definition, the estimated number of nonsmokers would increase by 2%, suggesting that the rate of misidentified occasional smokers among the self-reported nonsmokers was low. Second, people can be exposed to lead from residential, occupational, dietary, and other environmental sources (12). Information on some of these potential confounders (eg, hobbies involving metals) is not available for the NHANES population, but we were able to include age of residence and occupation, which are 2 of the most prominent sources of lead exposure. Third, the data are cross-sectional, making it impossible to determine whether tobacco smoke exposure preceded higher BLLs. As with some other chemicals of public health concern, lead persists in the body, complicating efforts to determine source attribution. However, our findings are consistent with those of other studies (26–28) that similarly relied on cross-sectional data to examine the relationship between tobacco smoke exposure and lead level.

Our study shows that increased BLLs for youths and adults are related to tobacco smoke exposure above and beyond exposures from older homes and occupations. Both in-home lead exposure and SHS exposure (15) are more prevalent among lower-income families, which further supports the need for public education about the dangers of lead exposure and smoking and the benefits of smoke-free home rules (29).

## Figures and Tables

**Table:** 

**1. The activity supported the learning objectives.**				
**Strongly Disagree**				**Strongly Agree**
1	2	3	4	5
**2. The material was organized clearly for learning to occur.**				
**Strongly Disagree**				**Strongly Agree**
1	2	3	4	5
**3. The content learned from this activity will impact my practice.**				
**Strongly Disagree**				**Strongly Agree**
1	2	3	4	5
**4. The activity was presented objectively and free of commercial bias.**				
**Strongly Disagree**				**Strongly Agree**
1	2	3	4	5

## Post-Test Information

To obtain credit, you should first read the journal article. After reading the article, you should be able to answer the following, related, multiple-choice questions. To complete the questions (with a minimum 70% passing score) and earn continuing medical education (CME) credit, please go to http://www.medscape.org/journal/pcd. Credit cannot be obtained for tests completed on paper, although you may use the worksheet below to keep a record of your answers. You must be a registered user on Medscape.org. If you are not registered on Medscape.org, please click on the "Register" link on the right hand side of the website to register. Only one answer is correct for each question. Once you successfully answer all post-test questions you will be able to view and/or print your certificate. For questions regarding the content of this activity, contact the accredited provider, CME@medscape.net. For technical assistance, contact CME@webmd.net. American Medical Association's Physician's Recognition Award (AMA PRA) credits are accepted in the US as evidence of participation in CME activities. For further information on this award, please refer to http://www.ama-assn.org/ama/pub/about-ama/awards/ama-physicians-recognition-award.page. The AMA has determined that physicians not licensed in the US who participate in this CME activity are eligible for **AMA PRA Category 1 Credits™**. Through agreements that the AMA has made with agencies in some countries, AMA PRA credit may be acceptable as evidence of participation in CME activities. If you are not licensed in the US, please complete the questions online, print the AMA PRA CME credit certificate and present it to your national medical association for review.

## Post-Test Questions

### Article Title: Obesity, Mortality, and Life Years Lost Associated With Breast Cancer in Nonsmoking US Women, National Health Interview Survey, 1997-2000

**CME Questions**

1. You are seeing a brother and sister, ages 3 and 13 years, respectively, for their annual health examinations. You find out that both parents smoke cigarettes, but they try to smoke outside of the house most of the time. According to the results of the current study by Richter and colleagues, what should you consider regarding the relationship between secondhand smoke (SHS) exposure and blood lead levels (BLLs)?
  - SHS exposure did not significantly affect BLLs after adjustment for confounders
  - SHS exposure was only associated with increases in BLLs among children younger than 12 years
  - SHS exposure was only associated with increases in BLLs among children 5 years or older
  - SHS exposure was associated with increases in BLLs among children and adolescents
2. The 13-year-old admits that she has started to smoke cigarettes herself. What do the results of the current study say regarding the effects of combined active smoking and SHS exposure?
  - Active smoking among youth was not associated with higher BLL
  - SHS exposure did not increase the BLL beyond that associated with active smoking
  - SHS exposure was associated with even higher BLLs among smokers
  - SHS exposure failed to affect BLLs in any analysis
3. Which cohort of individuals had the ***highest*** BLLs in the current study?
  - Children between ages 3 and 11 years with SHS exposure
  - Adolescents with SHS exposure
  - Adolescents who actively smoked cigarettes
  - Older adult smokers
4. As you evaluate these siblings, what should you consider regarding the temporal trend in BLLs during the study period of the current research?
  - BLLs increased with time among adults and children
  - BLLs increased with time among children but remained stable among adults
  - BLLs increased with time among children but declined among adults
  - BLLs declined among children and adults

**Evaluation**
